# Supplementary material for: A Comprehensive Updated Review on Magnetic Nanoparticles in Diagnostics
Source: Nanomaterials (Basel). 2021 Dec 17;11(12):3432. doi: 10.3390/nano11123432 (PMC8706278; doi:10.3390/nano11123432)
Supplement: Supplementary file 1 [file nanomaterials-11-03432-s001.zip › nanomaterials-1468667-supplementary.pdf]

Supporting Information

A Comprehensive Updated Review on Magnetic Nanoparticles in Diagnostics

Pedro Farinha [1], João M. P. Coelho [2,*], Catarina Pinto Reis [1,2,*] and Maria Manuela Gaspar [1,*]

[1] Research Institute for Medicines, iMed.ULisboa, Faculty of Pharmacy, Universidade de Lisboa, Av. Prof. Gama Pinto, 1649-003 Lisboa, Portugal; pedro-farinha@campus.ul.pt

[2] Instituto de Biofísica e Engenharia Biomédica (IBEB), Faculdade de Ciências, Universidade de Lisboa, Campo Grande, 1749-016 Lisboa, Portugal;

* Correspondence: jmcoelho@fc.ul.pt (J.M.P.C.); catarinareis@ff.ulisboa.pt (C.P.R.); mgaspar@ff.ulisboa.pt (M.M.G.)

Table S1. A general description of the most popular methods used for the synthesis of MNPs.

Technique		General Description	Ref
Physical	Pulsed laser ablation	A metal precursor bulk material is submerged in a liquid solvent. Then, a high energy laser is focused onto the metal causing its ablation which produces a plasma plume at high temperature and pressure composed of target and solvent. They then react and initiate nucleation and nanoparticle growth forming a colloid of nanoparticles	[1–4]
	Laser-induced pyrolysis	A laser is used to heat a gaseous mixture of organometallic precursors. This causes the molecular decomposition of the metal reagents into vapor. The vaporized components initiate nucleation and growth to form nanoparticles	[2,3,5,6]
	Spray pyrolysis	A solution of metal salts and a reducing agent is sprayed into a reactor. The solvent then evaporates, the metal precipitates and suffers annealing due to high temperature (thermolysis) and eventually forming the nanoparticles	[5,7–9]
	Power ball milling	The metal bulk material is placed inside a high-energy mill alongside balls made from strong alloys. The mill is then rotated with intense speed and the balls grind the power into nanosized particles through collision between the balls or between the balls and the inner walls of the mill.<br>Two approaches exist, dry milling and wet milling, the latter including a solution with surfactants which help reduce particle size	[10–12]
	Electron beam lithography	An electron beam is emitted against a film composed of metallic material submerged in a solvent. This causes the metal to heat up and evaporate and produce the nanoparticles	[2]
Chemical	Co-precipitation	Aqueous solutions containing different metal salts (ex: Fe^{2+}/Fe^{3+}) are co-precipitated by adding a base, preferably under heat and anaerobic conditions forming the nanoparticles	[3,13–16]

Hydrothermal	A mixture of iron salts is dissolved in an aqueous solution which is placed inside a reactor or autoclave. The temperature and pressure are raised which promotes hydrolysis and oxidation of the iron salts to form the particle crystals	[9,16–19]
Microemulsion	Generally, two identical water in oil (w/o) microemulsions are prepared, although some studies have used o/w microemulsions successfully. The first microemulsion contains the metal salts and the second one contains a precipitating agent, both present in the aqueous phase. The two microemulsions are then carefully mixed allowing the iron salts to react with the precipitating agent. The dispersed aqueous phase acts as a nano/microreactor creating a confined environment for nucleation and controlled growth of the particles. The water microdroplets will experience a cycle of continuous collision, coalescence and breaking, which allows for the chemical reactions to occur between the reagents and form the precipitated nanoparticles	[14,17–21]
Sonochemical	Involves the exposure of organoiron precursors to intense ultrasound waves. This induces acoustic cavitation, which is the generation, growth and collapse of bubbles in a liquid. The iron precursors form a shell around the bubble and once it implodes the shell collapses into the bubble center, creating the nanoparticle	[9,13,19,22]
Thermal Decomposition	Iron organometallic precursors are thermally decomposed (around 300 to 350°C) within high boiling point organic solvents to form iron oxide crystals. Surfactants are commonly used as capping agents to stabilize the crystals and improve particle size control	[13,17,19]
Electrochemical Decomposition	Two electrodes connected through a battery are then submerged in an electrolyte solution made of iron ions. The anode, which contains iron metal, is oxidized from metal to iron cation species which are dissolved in the solution and afterwards reduced back to metal by the cathode, forming the particles	[19,23]
Sol-gel	Iron alkoxides are dissolved in an aqueous solvent.<br>Iron alkoxides react with water, acids or bases and suffer hydroxylation to form iron oxide nanoparticles. This process forms a sol (a colloid made from very small particles).<br>The sol then undergoes condensation and forms a gel.<br>The gel undertakes a drying step to evaporate the solvent and the iron oxide nanoparticles are obtained	[9,17,18]
Polyol Method	Metal salts are added to a polyol solvent (from a simple ethylene glycol to various molecular sizes of PEG). The polyols function both as a stabilizing agent and a reducing agent, as well as prevent particle aggregation. As heat is applied the polyols suffer oxidation into various ketone and aldehyde species which then induce the reduction of the dissolved iron ions into IONPs	[1,9,24]

Biological		The plant phytochemicals and the microbial enzymes have reducing and biomineralization properties often used to reduce metal salts into nanoparticles	[13,18,25–27]

Table S2. Most commonly used methods for the synthesis of MNPs, their advantages and disadvantages.

Technique		Advantages	Disadvantages	Observations	Ref
Physical	Pulsed laser ablation	Simple, fast and cost-effective<br>Synthesis of monodisperse (uniform size and shape) particles<br>Eco-friendly since the method does not require use of chemicals	Difficulty in controlling particle size<br>Particle clustering	Top-down approach<br>Important Factors: laser intensity, wavelength and diameter	[1–3]
	Laser-induced pyrolysis	Controlled particle size<br>Narrow size distribution<br>Easy to scale-up<br>High production rate<br>Good for producing well dispersed small sized particles	Complex process<br>Relatively expensive	Bottom-up approach<br>Important Factors: vapor pressure and vaporization temperature of the precursors	[2,3,5,28]
	Spray pyrolysis	Controlled particle size and shape<br>Production of small sized particles	Particles tend to form aggregates<br>Expensive equipment<br>Interferences can be caused by oxygen and other reactive species present in the reactor	Bottom-up approach<br>vapor pressure and vaporization temperature of the precursors	[16,28]
	Power ball milling	Good reproducibility of particle size	Time-consuming<br>Low efficiency<br>Difficult to control particle size distribution	Top-down approach<br>Important Factors: milling time and speed	[2,4,10–12]

		Small and crystalline nanoparticles can be obtained<br>Simple and low cost<br>Easy to scale-up	Particles tend to form aggregates (although it is essentially exclusive to dry milling)		
	Electron beam lithography	Well-controlled interparticle spacing<br>Production of small sized particles<br>High production rate	Requires expensive and highly complex machines<br>Difficulty in in large scale production	Top-down approach<br>Considered more effective than photolithography	[2,9,29]
Chemical	Co-precipitation	Simple, convenient and effective<br>Cost-effective and high yielding<br>Very reproducible<br>Easy to scale-up	Inappropriate for the synthesis of high untainted, precise stoichiometric phase<br>Low degree of crystallinity<br>Relatively large polydispersity<br>To obtain a narrow size distribution, some reaction parameters must be strictly assured Particles tend to aggregate due to their small size	Bottom-up approach<br>Most commonly used method<br>Important Factors: ratio of salts, pH and ionic strength of the solution	[3,9,13–15,18,19,28]
	Hydrothermal	Controlled particle size and shape<br>Uniform size distribution<br>Low cost<br>Relatively easy to scale-up<br>Highly crystalline nanoparticles<br>Monodisperse particles can be obtained with shorter reaction times<br>The high temperature and pressure improve the nucleation rate and speed up the growth of new particles, resulting in the formation of small sized particle	Requires high pressure and reaction temperature<br>Most of the times polydisperse samples are obtained<br>Difficult to obtain quality nanocrystals smaller than 10 nm with hydrophilic surface properties<br>Slow reaction kinetics independent from the temperature applied (although microwave heating has been proven to assist in increasing the crystallization kinetics	Bottom-up approach<br>Important Factors: temperature, pressure, concentration of precursors and reaction time	[3,9,13,14,18,19]
	Microemulsion	The use of simple equipment<br>Controlled particle size, shape and composition<br>Produces small sized particles with uniform properties	The particle's properties are negatively affected by the residual surfactants present<br>The limited reaction temperature results in low yields and IONPs with low crystallinity<br>Difficult to scale-up	Bottom-up approach<br>Important Factors: choice of precipitating agent, surfactant concentration and water-to-surfactant ratio	[3,9,13,14,16,17,20,28]

	Can be used in simple conditions (near ambient temperature and pressure)			
Sonochemical	Narrow particle size distribution<br>This method provides monodisperse nanoparticles with a variety of shapes under ambient conditions<br>Does not require high bulk temperatures or long reaction times<br>If the goal is to produce amorphous nanoparticles, the sonochemical method offers better particle shape control than most other methods<br>Quick and low cost compared to other methods<br>Simple, low cost and eco-friendly	Mechanism is not well understood<br>Because of the high cooling rate of cavitation, it is difficult to produce crystallized particles. Therefore, the obtained amorphous particles need to be further processed by heat-treatment after they have been synthesized<br>Low efficiency	Bottom-up approach<br>Important Factors: sonication time and power, choice of capping agent and precursor concentration	[3,9,16,19,22,28,30]
Thermal Decomposition	Lower working temperature<br>Use of simple equipment<br>Control over the particle size<br>Hydrophilic particles are created which facilitates functionalization	Difficult to scale-up<br>Since the reaction occurs at room temperature the particles tend to show poor crystallinity	Bottom-up approach<br>Important Factors: current density and distance between electrodes	[19,23]
Electrochemical Decomposition	Controlled particle size and internal composition<br>Good mixing uniformity<br>High reaction uniformity<br>Low synthesis temperature<br>Low cost<br>High production rate	High permeability<br>Weak bonding<br>Low wear resistance<br>Needs post-treatment step to purify the particles from by-product contaminants<br>Limited efficiency<br>High cost	Bottom-up approach<br>Important Factors: temperature, pH, the chosen solvent and the used concentration of salt precursors	[3,9,16,18,19,28]
Sol-gel	Controlled particle size<br>Narrow size distribution<br>Good particle crystallinity<br>Good dispersibility	Uses toxic non environmentally friendly reagents, such as chloroform, hexane and iron pentacarbonyl<br>Laborious purification steps<br>The resulting nanoparticles are hydrophobic, so in order	Bottom-up approach<br>Important Factors: reaction time, the reaction temperature and the precursor-to-surfactant ratio	[3,13,17–19,28]

			to obtain water-soluble and biocompatible particles an additional surface modification step is required<br>Requires high temperatures<br>High cost<br>Time-consuming (long reaction time)		
	Polyol Method	Controlled particle shape and size<br>Uniform particle size<br>Easy to scale-up<br>Synthesis of crystalline nanoparticles due to the application of heat<br>Synthesis of metallic NPs coated in polyols granting them greater resistance against hydrolysis and oxidation	Limited efficiency and high cost<br>Requires high temperatures<br>Time-consuming	Bottom-up approach<br>Important Factors: molecular weight of the chosen polyol, precursor concentration and reaction temperature	[3,9,13,14,28]
Biological		Use of eco-friendly, non-toxic solvents<br>High biocompatibility<br>Cost effective and can be employed under ambient conditions	Mechanism is not well understood<br>Only certain plants can be used in the synthesis of nanoparticles<br>Plants produce low quantities of secreted enzymes which leads to a decreased rate of synthesis<br>Very time-consuming due to long periods of time needed for culturing microorganisms<br>Poor control over size, shape and crystallinity<br>Difficulty in producing monodispersed suspensions	Bottom-up approach<br>Important Factors: pH, pO_2, pCO_2, redox potential and temperature	[2,13,14,16,18,25,28]

Table S3. Some representative examples of IONPs in clinical trials or approved for clinical use.

Active Substance	Trade Name	Short Name	Surface Coating	Clinical Situation	Applications	Ref
Ferumoxtran	Combidex®(USA)<br>Sinerem® (EU)	AMI-227	Dextran	Ongoing clinical trials	Lymph node imaging<br>Cell labelling<br>Blood pool agent<br>CNS imaging	[19,31–36]
Ferucarbotran/ Ferrixican	Resovist® (USA and EU)<br>Cliavist® (France)	SHU-555A	Carboxydextran	Approved for clinical use<br>Withdrawn from the market since 2009 (USA and EU) due to lack of users<br>Available only in certain countries (ex: Japan)	Liver imaging<br>Cell labelling	[19,31–33,35–37]
	Supravist™	SHU-555C	Carboxydextran	Under clinical trials	MRI angiography<br>Cell labelling	[31,34]
Ferumoxide	Feridex® (USA)<br>Endorem™ (EU)	AMI-25	Dextran	Approved for clinical use<br>Withdrawn from the market since 2008 due to lack of users	Liver imaging<br>Cell labelling	[19,31,32,34–36,38]
Ferumoxytol	Feraheme® (USA)<br>Rienso® (EU)	Code 7228	Carboxymethyldextran	Approved for clinical use<br>Withdrawn from the EU market since 2015 due to lack of users	Treatment of IDA in patients with CKD<br>MRI angiography<br>Lymph node imaging<br>Primary tumor imaging<br>Multiple Sclerosis	[19,31,33,36,37,39–41]
Feruglose	Clariscan™	NC100150	PEGylated starch	Ongoing clinical trials	MRI angiography	[19,31,36,42,43]
Ferumoxsil	Lumirem® (USA)<br>GastroMARK® (EU)	AMI-121	Siloxane	Approved for clinical use<br>Withdrawn from the market due to lack of users	Oral GI imaging<br>Liver imaging	[19,31–33,35,36]
Ferristene	Abdoscan®	-	Sulfonated poly(styrene-	Approved for clinical use	Oral GI imaging	[19,31,35,36,44]

			divinylbenzene) copolymer	Unavailable on the market due to lack of users		
-	-	VSOP-C184	Citrate	Ongoing clinical trials	MRI angiography	[33,45–47]
-	Sienna+®	-	Carboxydextran	Approved in the EU	Lymph node imaging in breast cancer	[33,48]

Table S4. Examples of commercially available IONPs used in magnetic separation.

Company	Products	Applications	Reference
Stemcell	EasySep®<br>RoboSep®<br>SteamSep®	CS [1]	www.stemcell.com
Chemicell	Simag<br>fluidMAG<br>geneMAG<br>mHPA	NAS [2], PP [3]<br>CS<br>NAS<br>NAS, PP	www.chemicell.com
Dexter	LifeSep®	NAS, CS	www.dextermag.com
Ocean NanoTech	SuperMag Beads<br>MonoMag Beads<br>PureBind Beads	NAS, PP, CS	www.oceannanotech.com
TurboBeads	TurboBeads	NAS	www.turbobeads.com
SEPMAG	Sepmag®	NAS, PP, CS	www.sepmag.eu
Merck	Estapor®<br>PureProteome™<br>MagPrep®	NAS, PP, CS<br>PP<br>NAS, CS	www.merckmillipore.com
Miltenyi Biotec	CliniMACS®<br>autoMACS®<br>MultiMACS™	CS<br>NAS, PP, CS	www.miltenyibiotec.com
Invitrogen	MagniSort™<br>DynaMag™<br>Melon™<br>Pierce™<br>GeneCatcher™	CS<br>PP<br>NAS	www.thermofisher.com/invitrogen
Cube Biotech	PureCube	PP	www.cube-biotech.com

[1] Cell Separation
[2] Nucleic Acid Separation
[3] Protein Purification

References

1. Bensebaa, F. *Wet Production Methods*; 2013; Vol. 19; ISBN 9780123695505.
2. Samrot, A. V.; Sahithya, C.S.; Selvarani A, J.; Purayil, S.K.; Ponnaiah, P. A review on synthesis, characterization and potential biological applications of superparamagnetic iron oxide nanoparticles. *Curr. Res. Green Sustain. Chem.* **2021**, *4*, 1–13, doi:10.1016/j.crgsc.2020.100042.
3. Arias, L.S.; Pessan, J.P.; Vieira, A.P.M.; De Lima, T.M.T.; Delbem, A.C.B.; Monteiro, D.R. Iron oxide nanoparticles for biomedical applications: A perspective on synthesis, drugs, antimicrobial activity, and toxicity. *Antibiotics* **2018**, *7*, 1–32, doi:10.3390/antibiotics7020046.
4. Umer, A.; Naveed, S.; Ramzan, N.; Rafique, M.S. Selection of a suitable method for the synthesis of copper nanoparticles. *Nano* **2012**, *7*, 1–18, doi:10.1142/S1793292012300058.
5. Laurent, S.; Forge, D.; Port, M.; Roch, A.; Robic, C.; Vander Elst, L.; Muller, R.N. Magnetic iron oxide nanoparticles: Synthesis, stabilization, vectorization, physicochemical characterizations and biological applications. *Chem. Rev.* **2008**, *108*, 2064–2110, doi:10.1021/cr068445e.
6. Bomatí-Miguel, O.; Tartaj, P.; Morales, M.P.; Bonville, P.; Golla-Schindler, U.; Zhao, X.Q.; Veintemillas-Verdaguer, S. Core-shell iron-iron oxide nanoparticles synthesized by laser-induced pyrolysis. *Small* **2006**, *2*, 1476–1483, doi:10.1002/smll.200600209.
7. Gavrilović, T. V.; Jovanović, D.J.; Dramićanin, M.D. Synthesis of multifunctional inorganic materials: From micrometer to nanometer dimensions. *Nanomater. Green Energy* **2018**, 55–81, doi:10.1016/B978-0-12-813731-4.00002-3.
8. Eslamian, M.; Ahmed, M.; Ashgriz, N. Modelling of nanoparticle formation during spray pyrolysis. *Nanotechnology* **2006**, *17*, 1674–1685, doi:10.1088/0957-4484/17/6/023.
9. Ali, A.; Zafar, H.; Zia, M.; Haq, I. ul; Phull, A.R.; Ali, J.S.; Hussain, A. Synthesis, characterization, applications, and challenges of iron oxide nanoparticles. *Nanotechnol. Sci. Appl.* **2016**, *9*, 49–67.
10. Fan, D.; Wang, Q.; Zhu, T.; Wang, H.; Liu, B.; Wang, Y.; Liu, Z.; Liu, X.; Fan, D.; Wang, X. Recent Advances of Magnetic Nanomaterials in Bone Tissue Repair. *Front. Chem.* **2020**, *8*, 1–15, doi:10.3389/fchem.2020.00745.
11. Wu, K.; Su, D.; Liu, J.; Saha, R.; Wang, J.P. Magnetic nanoparticles in nanomedicine: A review of recent advances. *Nanotechnology* **2019**, *30*, doi:10.1088/1361-6528/ab4241.
12. Arbain, R.; Othman, M.; Palaniandy, S. Preparation of iron oxide nanoparticles by mechanical milling. *Miner. Eng.* **2011**, *24*, 1–9, doi:10.1016/j.mineng.2010.08.025.
13. Majidi, S.; Sehrig, F.Z.; Farkhani, S.M.; Goloujeh, M.S.; Akbarzadeh, A. Current methods for synthesis of magnetic nanoparticles. *Artif. Cells, Nanomedicine Biotechnol.* **2016**, *44*, 722–734, doi:10.3109/21691401.2014.982802.
14. Gul, S.; Khan, S.B.; Rehman, I.U.; Khan, M.A.; Khan, M.I. A Comprehensive Review of Magnetic Nanomaterials Modern Day Theranostics. *Front. Mater.* **2019**, *6*, 1–15, doi:10.3389/fmats.2019.00179.
15. Popescu, R.C.; Andronescu, E.; Vasile, B.S. Recent advances in magnetite nanoparticle functionalization for nanomedicine. *Nanomaterials* **2019**, *9*, 1–31, doi:10.3390/nano9121791.
16. Hosu, O.; Tertis, M.; Cristea, C. Implication of Magnetic Nanoparticles in Cancer Detection, Screening and Treatment. *Magnetochemistry* **2019**, *5*, 55, doi:10.3390/magnetochemistry5040055.
17. Al-Rawi, N.N.; Anwer, B.A.; Al-Rawi, N.H.; Uthman, A.T.; Ahmed, I.S. Magnetism in drug delivery: The marvels of iron oxides and substituted ferrites nanoparticles. *Saudi Pharm. J.* **2020**, *28*, 876–887, doi:10.1016/j.jsps.2020.05.003.
18. Chen, Z.; Wu, C.; Zhang, Z.; Wu, W.; Wang, X.; Yu, Z. Synthesis, functionalization, and nanomedical applications of functional magnetic nanoparticles. *Chinese Chem. Lett.* **2018**, *29*, 1601–1608, doi:10.1016/j.cclet.2018.08.007.
19. Dadfar, S.M.; Roemhild, K.; Drude, N.I.; Stillfried, S. von; Knüchel, R.; Kiessling, F.; Twan, L. Iron Oxide Nanoparticles: Diagnostic, Therapeutic and Theranostic Applications. *Adv. Drug Deliv. Rev.* **2019**, *138*, 302–325, doi:10.1016/j.addr.2019.01.005.Iron.
20. Okoli, C.; Sanchez-Dominguez, M.; Boutonnet, M.; Järås, S.; Civera, C.; Solans, C.; Kuttuva, G.R. Comparison and functionalization study of microemulsion-prepared magnetic iron oxide nanoparticles. *Langmuir* **2012**, *28*, 8479–8485, doi:10.1021/la300599q.

21. Bumajdad, A.; Ali, S.; Mathew, A. Characterization of iron hydroxide/oxide nanoparticles prepared in microemulsions stabilized with cationic/non-ionic surfactant mixtures. *J. Colloid Interface Sci.* **2011**, *355*, 282–292, doi:10.1016/j.jcis.2010.12.022.
22. Yadav, V.K.; Ali, D.; Khan, S.H.; Gnanamoorthy, G.; Choudhary, N.; Yadav, K.K.; Thai, V.N.; Hussain, S.A.; Manhrdas, S. Synthesis and characterization of amorphous iron oxide nanoparticles by the sonochemical method and their application for the remediation of heavy metals from wastewater. *Nanomaterials* **2020**, *10*, 1–17, doi:10.3390/nano10081551.
23. Cabrera, L.; Gutierrez, S.; Menendez, N.; Morales, M.P.; Herrasti, P. Magnetite nanoparticles: Electrochemical synthesis and characterization. *Electrochim. Acta* **2008**, *53*, 3436–3441, doi:10.1016/j.electacta.2007.12.006.
24. Eid, K.; Wang, H.; Wang, L. *Nanoarchitectonic Metals*; Elsevier Inc., 2017; ISBN 9780323378307.
25. Narayanan, K.B.; Sakthivel, N. Biological synthesis of metal nanoparticles by microbes. *Adv. Colloid Interface Sci.* **2010**, *156*, 1–13, doi:10.1016/j.cis.2010.02.001.
26. Ash, A.; Revati, K.; Pandey, B.D. Microbial synthesis of iron-based nanomaterials - A review. *Bull. Mater. Sci.* **2011**, *34*, 191–198, doi:10.1007/s12034-011-0076-6.
27. Asmathunisha, N.; Kathiresan, K. A review on biosynthesis of nanoparticles by marine organisms. *Colloids Surfaces B Biointerfaces* **2013**, *103*, 283–287, doi:10.1016/j.colsurfb.2012.10.030.
28. Ajinkya, N.; Yu, X.; Kaithal, P.; Luo, H.; Somani, P.; Ramakrishna, S. Magnetic iron oxide nanoparticle (IONP) synthesis to applications: Present and future. *Materials (Basel).* **2020**, *13*, 1–35, doi:10.3390/ma13204644.
29. Kurapov, Y.A.; Vazhnichaya, E.M.; Litvin, S.E.; Romanenko, S.M.; Didikin, G.G.; Devyatkina, T.A.; Mokliak, Y. V.; Oranskaya, E.I. Physical synthesis of iron oxide nanoparticles and their biological activity in vivo. *SN Appl. Sci.* **2019**, *1*, 1–11, doi:10.1007/s42452-018-0110-z.
30. Dheyab, M.A.; Aziz, A.A.; Jameel, M.S. Recent advances in inorganic nanomaterials synthesis using sonochemistry: A comprehensive review on iron oxide, gold and iron oxide coated gold nanoparticles. *Molecules* **2021**, *26*, 2–19, doi:10.3390/molecules26092453.
31. Jin, R.; Lin, B.; Li, D.; Ai, H. Superparamagnetic iron oxide nanoparticles for MR imaging and therapy: Design considerations and clinical applications. *Curr. Opin. Pharmacol.* **2014**, *18*, 18–27, doi:10.1016/j.coph.2014.08.002.
32. Vakili-Ghartavol, R.; Momtazi-Borojeni, A.A.; Vakili-Ghartavol, Z.; Aiyelabegan, H.T.; Jaafari, M.R.; Rezayat, S.M.; Arbabi Bidgoli, S. Toxicity assessment of superparamagnetic iron oxide nanoparticles in different tissues. *Artif. Cells, Nanomedicine Biotechnol.* **2020**, *48*, 443–451, doi:10.1080/21691401.2019.1709855.
33. Wáng, Y.X.J.; Idée, J.M. A comprehensive literatures update of clinical researches of superparamagnetic resonance iron oxide nanoparticles for magnetic resonance imaging. *Quant. Imaging Med. Surg.* **2017**, *7*, 88–122, doi:10.21037/qims.2017.02.09.
34. Oude Engberink, R.D.; Van Der Pol, S.M.A.; Walczak, P.; Van Der Toorn, A.; Viergever, M.A.; Dijkstra, C.D.; Bulte, J.W.M.; De Vries, H.E.; Blezer, E.L.A. Magnetic resonance imaging of monocytes labeled with ultrasmall superparamagnetic particles of iron oxide using magnetoelectroporation in an animal model of multiple sclerosis. *Mol. Imaging* **2010**, *9*, 268–277, doi:10.2310/7290.2010.00016.
35. Norouzi, M.; Derakhshanfar, S.; Kazemian, M.R.; França, R. Superparamagnetic Iron Oxide Nanoparticles in Cancer Theranostics. **2018**, *2*, 1–6.
36. Li, X.; Sun, Y.; Ma, L.; Liu, G.; Wang, Z. The Renal Clearable Magnetic Resonance Imaging Contrast Agents: State of the Art and Recent Advances. *Molecules* **2020**, *25*, 1–16, doi:10.3390/molecules25215072.
37. Thakor, A.S.; Jokerst, J. V.; Ghanouni, P.; Campbell, J.L.; Mittra, E.; Gambhir, S.S. Clinically Approved Nanoparticle Imaging Agents. *J. Nucl. Med.* **2016**, *57*, 1833–1837, doi:10.2967/jnumed.116.181362.
38. Modo, M.; Kolosnjaj-Tabi, J.; Nicholls, F.; Ling, W.; Wilhelm, C.; Debarge, O.; Gazeau, F.; Clement, O. Considerations for the clinical use of contrast agents for cellular MRI in regenerative medicine. *Contrast Media Mol. Imaging* **2013**, *8*, 439–455, doi:10.1002/cmmi.1547.
39. AMAG Pharmaceuticals and Takeda Announce Mutual Termination of Agreement to License, Develop and Commercialize Ferumoxytol in Ex-U.S. Territories, Including Europe Available online: https://www.takeda.com/newsroom/newsreleases/2015/amag-pharmaceuticals-and-takeda-announce-mutual-termination-of-agreement-to-license-develop-and-commercialize-ferumoxytol-in-ex-u.s.-

territories-including-europe/ (accessed on Jul 11, 2021).
40. European Medicines Agency: EMA/437901/2015 - Rienso, withdrawal of the marketing authorisation in the European Union.
41. European Medicines Agency: EMA/102191/2015 - EPAR summary for the public Rienso Available online: https://www.ema.europa.eu/en/documents/overview/rienso-epar-summary-public_en.pdf (accessed on Jul 11, 2021).
42. Leiner, T.; Ho, K.Y.J.A.M.; Ho, V.B.; Bongartz, G.; Mali, W.P.T.M.; Engelshoven, J.M.A. van Multicenter phase-II trial of safety and efficacy of NC100150 for steady-state contrast-enhanced peripheral magnetic resonance angiography. *Eur. Radiol.* **2003**, *13*, 1620–1627.
43. Weishaupt, D.; Rühm, S.G.; Binkert, C.A.; Schmidt, M.; Patak, M.A.; Steybe, F.; McGill, S.; Debatin, J.F. Equilibrium-phase MR angiography of the aortoiliac and renal arteries using a blood pool contrast agent. *Am. J. Roentgenol.* **2000**, *175*, 189–195, doi:10.2214/ajr.175.1.1750189.
44. Schneider, M.G.M.; Lassalle, V.L. Magnetic iron oxide nanoparticles as novel and efficient tools for atherosclerosis diagnosis. *Biomed. Pharmacother.* **2017**, *93*, 1098–1115, doi:10.1016/j.biopha.2017.07.012.
45. Taupitz, M.; Wagner, S.; Schnorr, J.; Kravec, I.; Pilgrimm, H.; Bergmann-Fritsch, H.; Hamm, B. Phase I clinical evaluation of citrate-coated monocrystalline very small superparamagnetic iron oxide particles as a new contrast medium for magnetic resonance imaging. *Invest. Radiol.* **2004**, *39*, 394–405, doi:10.1097/01.rli.0000129472.45832.b0.
46. Wagner, M.; Wagner, S.; Schnorr, J.; Schellenberger, E.; Kivelitz, D.; Krug, L.; Dewey, M.; Laule, M.; Hamm, B.; Taupitz, M. Coronary MR angiography using citrate-coated very small superparamagnetic iron oxide particles as blood-pool contrast agent: Initial experience in humans. *J. Magn. Reson. Imaging* **2011**, *34*, 816–823, doi:10.1002/jmri.22683.
47. Schnorr, J.; Wagner, S.; Abramjuk, C.; Wojner, I.; Schink, T.; Kroencke, T.J.; Schellenberger, E.; Hamm, B.; Pilgrimm, H.; Taupitz, M. Comparison of the iron oxide-based blood-pool contrast medium VSOP-C184 with gadopentetate dimeglumine for first-pass magnetic resonance angiography of the aorta and renal arteries in pigs. *Invest. Radiol.* **2004**, *39*, 546–553, doi:10.1097/01.rli.0000133944.30119.cc.
48. Teshome, M.; Wei, C.; Hunt, K.K.; Thompson, A.; Rodriguez, K.; Mittendorf, E.A. Use of a Magnetic Tracer for Sentinel Lymph Node Detection in Early-Stage Breast Cancer Patients: A Meta-analysis. *Ann. Surg. Oncol.* **2016**, *23*, 1508–1514, doi:10.1245/s10434-016-5135-1.
